# The association of county-level socioeconomic factors with individual tobacco and alcohol use: a longitudinal study of U.S. adults

**DOI:** 10.1186/s12889-019-6700-x

**Published:** 2019-04-11

**Authors:** Rita Hamad, Daniel M. Brown, Sanjay Basu

**Affiliations:** 1 Department of Family and Community Medicine, Philip R. Lee Institute for Health Policy Studies, University of California San Francisco, 995 Potrero Avenue, Building 80, Ward 83, San Francisco, CA 94110 USA; 2 Environmental Health Sciences Division, University of California Berkeley, Berkeley, California USA; 3 Department of Medicine, Stanford University, Palo Alto, California USA

**Keywords:** Alcohol, Tobacco, Area effects, County effects, Fixed effects, Health disparities, Social epidemiology

## Abstract

**Background:**

Place-based factors have been implicated as root causes of socioeconomic disparities in risky health behaviors such as tobacco and alcohol use. Yet few studies examine the effects of county-level socioeconomic characteristics, despite the fact that social and public health policies are often implemented at the county level. In this study, we tested the hypothesis that county-level socioeconomic disadvantage was associated with individual tobacco and alcohol use.

**Methods:**

The sample included a panel of participants from the National Longitudinal Survey of Youth (*N* = 9302). The primary predictors were three time-varying measures of socioeconomic disadvantage in an individual’s county of residence: educational attainment, percent unemployment, and per capita income. We first conducted traditional ordinary least squares (OLS) models, both unadjusted and adjusted for individual-level covariates. We then conducted fixed effects (FE) models to adjust for confounding by unmeasured time-invariant individual-level factors.

**Results:**

OLS and FE models yielded contrasting results: higher county-level per capita income was associated with decreased drinking in OLS models and increased drinking in FE models, while decreased county-level educational attainment was associated with decreased smoking in OLS models and more cigarettes per day in FE models. The findings from FE models suggest that OLS models were confounded by unobserved time-invariant characteristics. Notably, the point estimates for the county-level measures were small, and in many cases they may not represent a clinically meaningful effect except at the population level.

**Conclusions:**

These results suggest that county-level socioeconomic characteristics may modestly influence tobacco and alcohol use. Future work should examine the effects of specific county policies that might explain these findings.

**Electronic supplementary material:**

The online version of this article (10.1186/s12889-019-6700-x) contains supplementary material, which is available to authorized users.

## Background

**Fig. 1:**
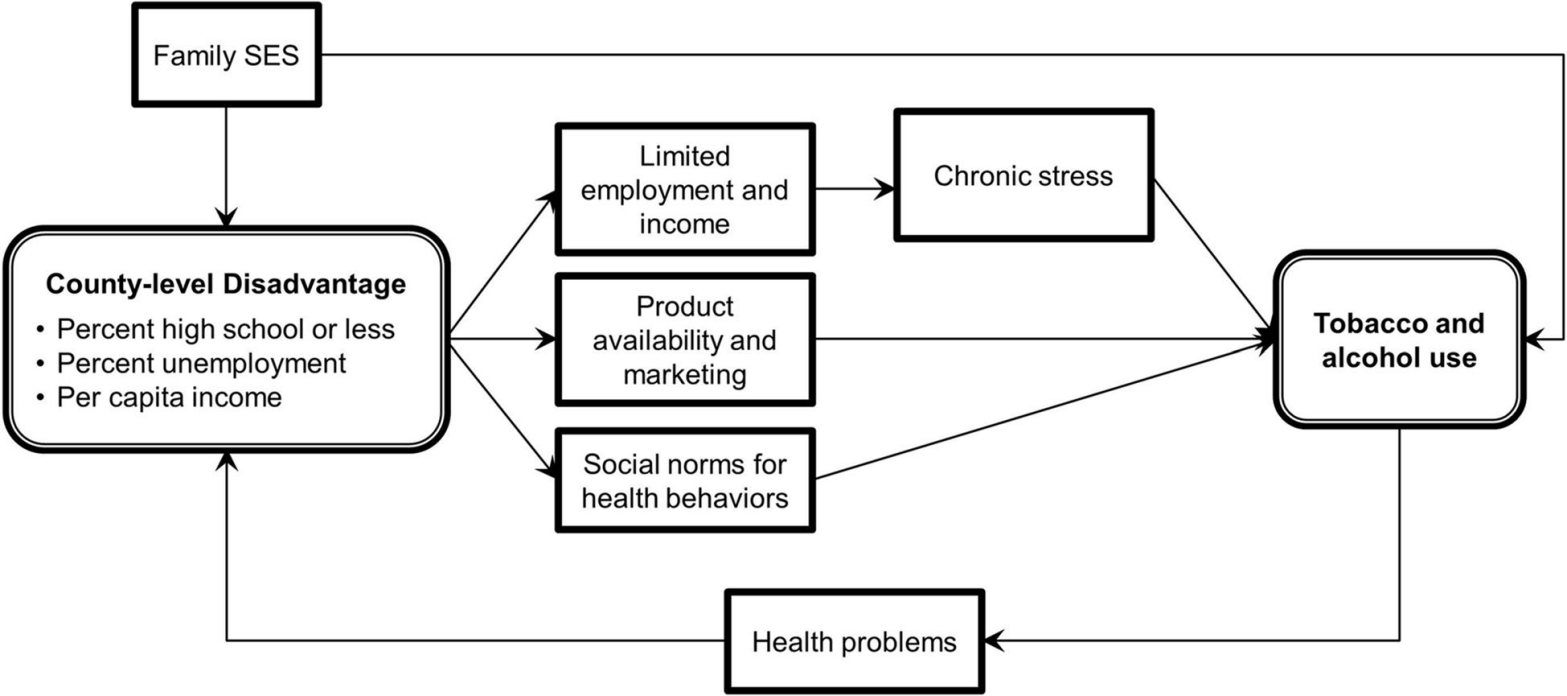
Conceptual model linking neighborhood socioeconomic status with tobacco and alcohol use

Place-based characteristics have been implicated as determinants of socioeconomic disparities in risky health behaviors, over and above the effects of individual-level socioeconomic status. For example, numerous studies have demonstrated associations between area-level disadvantage—including measures of poverty, education, employment, or an aggregate index of all three—and tobacco and alcohol use [1–5]. Hypothesized mechanisms linking area-level disadvantage with healthy risk behaviors include limited employment and income, leading to stress and increased substance use [1, 6], the availability of harmful substances, for example through the increased marketing of tobacco products in low-income areas [7, 8], or differences in social norms [9–11] (Fig. 1). Area-level policies—such as taxation or smoking restrictions—may also drive differences in the prevalence of substance use [12–15].

While numerous prior studies have examined the health effects of small areas (e.g., neighborhoods or U.S. census tracts) and large areas (e.g., states, countries) [16–21], few have examined the effects of U.S. county-level characteristics on risky health behaviors [20, 22]. There are roughly 3000 counties in the U.S., and they represent administrative areas that are larger than neighborhoods but smaller than states. Analyses at the county level are important because relevant policies that influence health and the social determinants of health are often implemented by county governments [23]. Examples include county-level “smoke-free” policies that restrict smoking in certain places [24–26], those that limit the sale of specific tobacco products [27, 28], and others that limit the sale of alcohol [29]. Beyond just health policy, counties are also involved in policies that affect the *social and economic determinants* of health behaviors. For example, income is strongly correlated with tobacco and alcohol use [30, 31], and counties are often involved in policies that affect labor markets or other economic factors, like setting a local minimum wage [32].

A recent review concluded that the evidence on the associations between area-level characteristics and individual health behaviors remains inconclusive [33]. In part, prior work may demonstrate inconsistent results because of the use of different measures of disadvantage [33]. More problematically, some prior analyses may suffer from confounding or reverse causation, in that unhealthy individuals may be more likely to move into disadvantaged areas [34, 35]. Simple adjustment for observed individual-level covariates is unlikely to adequately control for this confounding, such that more rigorous study designs may be needed. Previous studies using more sophisticated statistical methods—including fixed effects (FE) and marginal structural models—find persistent associations between area-level deprivation and tobacco and alcohol use [36, 37]. Yet these studies were not conducted using population-level U.S. data, which limits their generalizability, and they employed limited measures of area-level disadvantage.

In this study, we estimated the association between county-level characteristics and health behaviors using FE models, which more rigorously adjust for confounding relative to standard statistical techniques used in the prior literature. We employed a large nationally representative U.S. sample to test the hypothesis that greater county-level socioeconomic disadvantage is associated with increased risky health behaviors, even after adjusting for individual-level socioeconomic status. In addition, we examined multiple indices of area-level disadvantage. Determining the contributions of county-level socioeconomic characteristics to disparities in risky health behaviors has important implications for directing policy budgets towards effective interventions.

## Methods

### Data set

We used data from the 1979 National Longitudinal Survey of Youth (NLSY). The NLSY is a nationally representative longitudinal panel study of 12,686 men and women in the United States enrolled when they were 14–22 years old in 1979. It was conducted annually during 1979–1994 and biennially thereafter, via in-person interviews. Questions regarding the health outcomes of interest were included in surveys beginning in 1992 for smoking, and in 1994 for alcohol use. We restricted the sample to individuals who answered questions related to the health outcomes of interest in at least the first time period, and who lived in counties for which county-level socioeconomic data were available. This resulted in a sample of 9302 individuals in 2117 counties. Additional details on the NLSY are provided elsewhere [38].

### Individual-level covariates

Time-invariant characteristics included gender and race. Time-varying covariates included educational attainment, marital status, number of children in the household, annual total household income in inflation-adjusted U.S. dollars, and the number of weeks of unemployment in the last year. For the latter two variables, the natural logarithm was taken because of right-skewness. All models also included fixed effects (i.e., indicator variables) for year to account for secular trends.

### County-level disadvantage

**Fig. 2:**
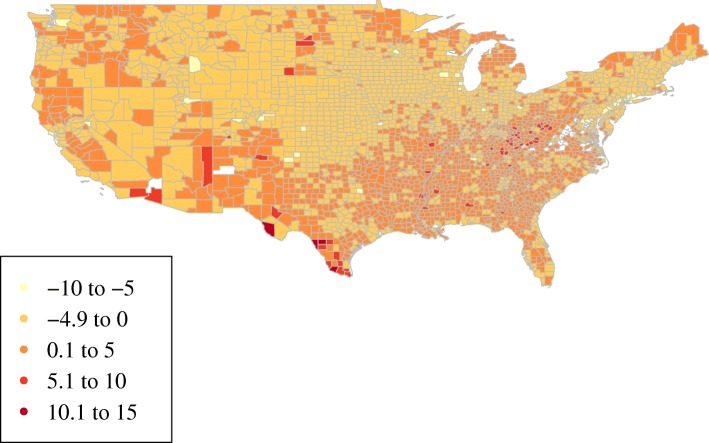
County socioeconomic disadvantage, 1992. Higher values represent higher levels of county socioeconomic disadvantage. For illustrative purposes, measures of county-level educational attainment, unemployment, and income were standardized with a mean of zero and standard deviation of one, and these three values were then summed to obtain the composite index shown here. Source: Authors’ calculations using publicly available data from the U.S. Bureau of Labor Statistics, the Bureau of Economic Analysis, and the Census Bureau

We constructed three variables to capture the level of disadvantage in each individual’s county of residence in a given year: (1) educational attainment, i.e., the percent of people in a county with a high school education or less, (2) percent unemployment, and (3) inflation-adjusted per capita personal income. Each of these has been previously associated with substance use in correlational studies [2, 37, 39, 40]. These measures were obtained from online national public data sources [41–44]. The three time-varying exposure variables were then linked to NLSY respondents based on their county of residence during each survey wave. Figure 2 shows the variation in county-level disadvantage in 1992, the beginning of the study period.

### Health behavior outcomes

Two alcohol-related outcomes were constructed using NLSY survey questions (Table 1), including the number of alcoholic drinks consumed in a typical day in the last month, and whether an individual consumed at least six drinks in a single day in the last month. We refer to the latter as binge drinking for conciseness, as it roughly corresponds to the term established by the U.S. National Institute on Alcohol Abuse and Alcoholism: four or more drinks per day for women and five or more drinks for men [45]. The NLSY does not contain a question that captures this standard definition of binge drinking. We also constructed two smoking-related variables, including the number of cigarettes smoked per day in the last month, and whether an individual was a current smoker in the last month.

**Table 1:** Health Outcomes of Interest, U.S. National Longitudinal Study of Youth, 1992–2012

Variable	Type	Survey Years Including Outcome
Binge drinking in last month	Binary	1994, 2002, 2006, 2008, 2010, 2012
Typical number of drinks per day (zero for non-drinkers)	Continuous	1994, 2002, 2006, 2008, 2010, 2012
Smoked in last month	Binary	1992, 1994, 1998, 2008, 2010, 2012
Typical number of cigarettes per day (zero for non-smokers)	Continuous	1992, 1994, 1998, 2008, 2010, 2012

### Multiple imputation

We conducted multiple imputation using chained equations to impute missing predictor variables from the NLSY. The percentage of missing values ranged from roughly 2% for weeks of unemployment to about 30% for household income. We assumed that values were “missing at random,” rather than “missing completely at random” [46]. This imputation method does not assume that variables are normally distributed, and can therefore be employed for categorical and binary variables. The data were imputed in wide form, to allow for correlations between observations of the same individual in different years. All variables used in the analytic models were included in the imputation models, including outcome variables, in order to improve the prediction of missing covariates. We did not use imputed values of the outcome variables in the analyses, however, as this is likely to add noise to subsequent estimates [47]. This resulted in differing numbers of observations for analyses examining each of the different health outcomes. We produced 20 imputations per observation, which is sufficient to ensure reproducibility between successive analytic runs [48].

As a sensitivity analysis, we also conducted the models described below using the complete cases, i.e., excluding observations with missing values.

### Data analysis

We employed two types of models in this study. First, we conducted standard ordinary least squares (OLS) models to examine the association between health behaviors and county-level disadvantage. We then carried out individual-level fixed effects (FE) models, which adjust for time-invariant confounding and therefore captured the effects of “within-person” changes in county-level disadvantage. FE models represent an improvement over OLS models in that they compare each individual with herself at different time points, rather than comparing different individuals to one another. This amounts to adding a separate intercept for each individual, thereby controlling for any unobserved characteristics that are constant over time [49]. The main drawback of FE methods is that they rely on multiple observations per person; studies that only include a single measurement for a given individual cannot leverage this technique. In this study, we employed both techniques to investigate whether methodological differences may explain heterogeneity in the prior literature.

Logistic regressions with FE were not feasible due to the sheer number of parameters and the failure of these models to converge. We therefore report the results of linear regressions for continuous outcomes as well as binary outcomes (i.e., linear probability models). As a sensitivity analysis, we carried out logistic regressions for binary outcomes in the OLS models, and these resulted in findings that were similar in magnitude and statistical significance to our primary findings (results available upon request).

#### Ordinary least squares models

We first conducted multivariable regressions to examine the association between each of the four outcome variables and the three measures of county-level disadvantage. We fit two sets of models: the first included only the three measures of disadvantage (unadjusted), while the second also included the time-variant and time-invariant individual-level covariates listed above (adjusted).

Because standard errors between observations may be correlated over time, we employed Huber-White robust standard errors clustered at the individual level to account for potential heteroscedasticity [50], analogous to generalized estimating equations. Multi-level models (also known as hierarchical models) are primarily useful when the question of interest is decomposition of the variance at multiple levels of analysis [51], which was not our research question of interest.

#### Fixed effects models

We next conducted multivariable linear regressions, now with the inclusion of FE at the individual level. This accounted for confounding by unmeasured time-invariant characteristics of the individual and their contemporaneous county of residence. We carried out two sets of models, with and without adjusting for the time-varying individual-level covariates listed above. Robust standard errors were again clustered at the individual level to account for correlated observations.

#### Secondary analysis

Because of potential lagged effects of county-level socioeconomic characteristics on health behaviors, we also carried out an analysis in which the primary exposures were unemployment rates, per capita income, and educational attainment in an individual’s county-of-residence during the *prior* survey wave. We conducted these analyses using OLS and FE models. These analyses were otherwise similar to our primary models, including adjustment for covariates and clustering of standard errors.

## Results

### Sample characteristics

The sample was diverse with respect to gender, educational attainment, and race (Table 2). The sociodemographic characteristics of those living in socioeconomically disadvantaged counties were statistically significantly different from those living in non-disadvantaged counties. Those in disadvantaged counties were more likely to be female, non-white, and unmarried, and were more likely to have lower educational attainment, lower household income, and more weeks unemployed in the last year. In terms of health behaviors, those living in disadvantaged counties smoked fewer cigarettes on average. They were less likely to be binge drinkers, and consumed fewer drinks per day.

**Table 2:** Sample Characteristics by County Disadvantage Level, U.S. National Longitudinal Study of Youth, 1992–2012 (*N* = 9302)

	County Not Socioeconomically Disadvantaged	County Socioeconomically Disadvantaged	Test of proportions or t-test (*p*-value)
Sociodemographic Characteristics			
Female (%)	51.2	51.9	<0.01
Education (%)			
Less than high school	14.00	18.8	<0.01
High school	36.3	40.4	<0.01
Some college	24.7	25.0	<0.01
College or more	24.9	15.7	<0.01
Race (%)			
White/other	52.8	41.5	<0.01
Black	30.9	32.4	<0.01
Hispanic	16.3	26.1	<0.01
Married (%)	66.5	64.4	<0.01
Children in household (mean (SD))	1.2 (1.2)	1.2 (1.3)	0.20
Annual household income (USD, mean (SD))	59,956 (61,500)	49,119 (53,005)	<0.01
Weeks unemployed in last year (mean (SD))	2.3 (8.2)	3.0 (9.7)	<0.01
Health Behaviors			
Tobacco-related outcomes			
Smoker (%)	30.0	30.2	0.16
Cigarettes per day (mean (SD))	4.2 (8.6)	4.1 (8.7)	<0.01
Alcohol-related outcomes			
Binge drinker (%)	18.4	17.8	<0.01
Drinks per day (mean (SD))	1.8 (2.3)	1.7 (2.5)	<0.01

Note: Values for sociodemographic characteristics are based on imputed data. Income was inflation-adjusted. For illustrative purposes to summarize sample characteristics, measures of county-level disadvantage (i.e., educational attainment, unemployment, and income) were each standardized with a mean of zero and standard deviation of one, these three values were summed to obtain a composite index, and this index was then split at the median

### Ordinary least squares models

For unadjusted OLS models (Table 3), increased county-level unemployment was associated with decreased smoking, fewer cigarettes per day, and more drinks per day. Increased county-level per capita income was associated with decreased smoking, fewer cigarettes per day, and less binge drinking. Lower county-level educational attainment was associated with less smoking. Results were largely similar in adjusted OLS models (Table 3), although unemployment was no longer statistically significantly associated with drinks per day.

**Table 3:** Ordinary Least Squares Analysis of the Association between County-Level Characteristics and Individual Health Behaviors, U.S. National Longitudinal Study of Youth, 1992–2012

	β Coefficient [95% CI]							
	Smoker Unadjusted	Smoker Adjusted	Daily Cigarettes Unadjusted	Daily Cigarettes Adjusted	Binge Drinking Unadjusted	Binge Drinking Adjusted	Daily Drinks Unadjusted	Daily Drinks Adjusted
County characteristics								
Unemployment rate	− 0.0055*	− 0.0053*	− 0.14*	− 0.085*	− 0.00064	− 0.0011	0.021*	0.012
	[−0.0080, − 0.0031]	[− 0.0077, − 0.0029]	[− 0.19, − 0.094]	[− 0.13, − 0.039]	[− 0.0031, 0.0018]	[− 0.0036, 0.0014]	[0.0037, 0.037]	[− 0.0055, 0.030]
Income (per $1000)	− 0.0020*	− 0.00080*	− 0.055*	− 0.030*	− 0.00088*	− 0.00070*	− 0.0025	− 0.0019
	[−0.0028, − 0.0013]	[− 0.0015, − 0.00009]	[− 0.067, − 0.042]	[− 0.042, − 0.018]	[− 0.0015, − 0.00031]	[− 0.0013, − 0.00014]	[−0.0055, 0.00054]	[− 0.0049, 0.0010]
% < high school	− 0.00090*	− 0.00099*	− 0.0085	−0.0014	0.00025	0.00024	−0.00017	0.000077
	[−0.0016, − 0.00023]	[− 0.0017, − 0.00028]	[− 0.019, 0.0019]	[− 0.012, 0.0098]	[− 0.00032, 0.00081]	[− 0.00036, 0.00083]	[− 0.0033, 0.0029]	[− 0.0032, 0.0033]
Female		−0.021*		−0.67*		−0.15*		−0.83*
		[−0.037, − 0.0051]		[− 0.97, − 0.38]		[− 0.17, − 0.14]		[− 0.90, − 0.77]
Race (ref white/other)								
Black		−0.048*		−2.76*		−0.066*		−0.52*
		[−0.067, − 0.029]		[− 3.12, − 2.40]		[− 0.079, − 0.053]		[−0.59, − 0.44]
Hispanic		−0.12*		−3.64*		−0.015		0.015
		[−0.14, − 0.096]		[−4.03, − 3.25]		[− 0.032, 0.0018]		[− 0.086, 0.12]
Ln(Household income)		−0.0087*		−0.074*		0.0063*		0.055*
		[−0.012, − 0.0057]		[− 0.12, − 0.025]		[0.0045, 0.0080]		[0.044, 0.066]
Education (ref < HS)								
High school		−0.15*		−2.84*		−0.013		−0.16*
		[−0.18, − 0.13]		[− 3.41, − 2.28]		[− 0.031, 0.0046]		[− 0.28, − 0.030]
Some college		−0.22*		−4.02*		−0.054*		−0.36*
		[−0.24, − 0.19]		[−4.58, − 3.47]		[− 0.073, − 0.034]		[−0.49, − 0.24]
College or more		−0.36*		−6.57*		−0.092*		−0.54*
		[−0.39, − 0.33]		[−7.13, − 6.00]		[− 0.11, − 0.072]		[−0.67, − 0.41]
Married		−0.074*		−0.96*		−0.053*		−0.37*
		[−0.094, − 0.055]		[− 1.30, − 0.62]		[−0.068, − 0.039]		[− 0.45, − 0.29]
Ln(Weeks unemployed)		0.036*		0.62*		0.011*		0.090*
		[0.030, 0.043]		[0.49, 0.74]		[0.0063, 0.016]		[0.058, 0.12]
Number of children		−0.016*		−0.26*		−0.0097*		−0.035*
		[−0.022, − 0.011]		[− 0.37, − 0.16]		[−0.014, − 0.0052]		[− 0.062, − 0.0092]
No. Observations	42,996	42,996	40,735	40,735	39,402	39,402	44,272	44,272
No. Individuals	9246	9246	9206	9206	8943	8943	9064	9064

*p < 0.05. HS = high school. County-level characteristics included annual unemployment rate, inflation-adjusted per capita personal income, and percent with less than a high school education. Analyses were conducted using multivariable linear regressions with imputed data. Linear probability models were used for binary outcomes. Additional controls included fixed effects for year

Analyses using complete cases yielded results similar to those obtained with imputed data (results available upon request).

### Fixed effects models

In unadjusted FE models (Table 4), increased county-level unemployment was associated with decreased smoking, fewer cigarettes per day, and more drinks per day. Increased county-level per capita income was associated with higher rates of binge drinking and more drinks per day (both contradictory to OLS findings). Results were similar in adjusted FE models for unemployment, and additionally, lower county-level educational attainment was associated with more cigarettes per day (again contradictory to OLS findings).

**Table 4:** Fixed Effects Analysis of the Association between County-Level Characteristics and Individual Health Behaviors, U.S. National Longitudinal Study of Youth, 1992–2012

	β Coefficient [95% CI]							
	Smoker Unadjusted	Smoker Adjusted	Daily Cigarettes Unadjusted	Daily Cigarettes Adjusted	Binge Drinking Unadjusted	Binge Drinking Adjusted	Daily Drinks Unadjusted	Daily Drinks Adjusted
County characteristics								
Unemployment rate	−0.0036*	− 0.0034*	− 0.056*	− 0.053*	− 0.000045	0.00020	0.032*	0.026*
	[− 0.0060, − 0.0012]	[−0.0058, − 0.00092]	[−0.098, − 0.013]	[−0.098, − 0.0084]	[−0.0030, 0.0029]	[− 0.0029, 0.0033]	[0.0082, 0.055]	[0.0025, 0.050]
Income (per $1000)	−0.00052	−0.00056	− 0.0049	−0.0069	0.00095*	0.00077	0.0062*	0.0042
	[−0.0012, 0.00018]	[− 0.0013, 0.00017]	[− 0.017, 0.0074]	[−0.020, 0.0063]	[0.00008, 0.0018]	[−0.00013, 0.0017]	[0.00079, 0.012]	[−0.0013, 0.0097]
% < high school	− 0.00020	−0.00015	0.0068	0.0086*	− 0.000033	−0.000060	− 0.0020	−0.0015
	[−0.00072, 0.00032]	[−0.00070, 0.00040]	[− 0.0010, 0.015]	[0.00013, 0.017]	[− 0.00059, 0.00052]	[− 0.00065, 0.00053]	[−0.0053, 0.0012]	[− 0.0049, 0.0019]
Ln(Household income)		−0.00023		0.048*		0.0059*		0.050*
		[−0.0024, 0.0019]		[0.0057, 0.091]		[0.0039, 0.0079]		[0.037, 0.064]
Education (ref < HS)								
High school		−0.0015		−0.10		−0.0063		0.045
		[−0.025, 0.022]		[−0.57, 0.36]		[−0.029, 0.017]		[−0.16, 0.25]
Some college		0.000067		−0.14		−0.015		0.051
		[−0.027, 0.027]		[−0.68, 0.39]		[−0.042, 0.012]		[−0.17, 0.28]
College or more		0.0013		0.34		−0.0042		0.14
		[−0.035, 0.038]		[−0.34, 1.02]		[−0.043, 0.034]		[−0.14, 0.43]
Married		−0.031*		−0.49		−0.044*		−0.21*
		[−0.047, − 0.015]		[− 0.80, − 0.18]		[−0.065, − 0.022]		[− 0.34, − 0.085]
Ln(Weeks unemployed)		0.0060*		0.12*		0.0017		0.032*
		[0.0021, 0.0099]		[0.046, 0.20]		[−0.0030, 0.0064]		[0.00084, 0.064]
Number of children		−0.0016		0.066		0.0044		0.0094
		[−0.0062, 0.0030]		[−0.018, 0.15]		[−0.0013, 0.010]		[−0.026, 0.045]
No. Observations	42,996	42,996	40,735	40,735	39,402	39,402	44,272	44,272
No. Individuals	9246	9246	9206	9206	8943	8943	9064	9064

*p < 0.05. HS = high school. County-level characteristics included annual unemployment rate, inflation-adjusted per capita personal income, and percent with less than a high school education. Analyses were conducted using multivariable linear regressions with imputed data, including fixed effects at the individual level to adjust for time-invariant individual characteristics. Linear probability models were used for binary outcomes. Additional controls included fixed effects for year

Analyses using complete case data yielded results similar to those obtained with imputed data (results available upon request).

### Secondary analyses

For adjusted OLS models using lagged exposures (Additional file 1: Table S1), increased county-level unemployment was associated with decreased smoking and fewer cigarettes per day, as in our primary models. Increased county-level per capita income was associated with decreased smoking, fewer cigarettes per day, and less binge drinking, as in our primary models, as well as fewer drinks per day. Lower county-level educational attainment was associated with increased binge drinking and drinks per day, neither of which was statistically significant in our primary models.

In adjusted FE models using lagged exposures (Additional file 2: Table S2), increased county-level unemployment was associated with decreased smoking and fewer cigarettes per day, as in our primary models, although drinks per day was no longer statistically significant. Increased county-level per capita income was associated with higher rates of binge drinking as in our primary models, and drinks per day was no longer statistically significant. There was no association between county-level educational attainment and health behaviors.

## Discussion

In this study, we investigated how three measures of county-level socioeconomic disadvantage were associated with individual tobacco and alcohol use, using a large longitudinal nationally representative U.S. data set. In both OLS and FE models, higher unemployment rates were associated with less smoking and more drinks per day. Yet OLS and FE models gave contrasting results for the other county-level socioeconomic measures: higher county-level per capita income was associated with decreased drinking in OLS models and increased drinking in FE models, while decreased area-level educational attainment was associated with decreased smoking in OLS models and more cigarettes per day in FE models. Results for lagged models were similar, which may be because socioeconomic characteristics in a given county are correlated over time. The findings from the FE models suggest that OLS models are confounded by unobserved time-invariant individual-level characteristics. Of note, the point estimates for each of our analyses were very small, and in many cases may not represent a meaningful effect except at the population level.

These findings suggest that interventions to address the social and economic determinants of health at the population level may influence levels of tobacco and alcohol use, thereby improving population health. Prior work has shown that policies at the state level in the U.S. are associated with improvements in child health and chronic disease [17, 52–54], although research on county-level policies is limited [32]. Future studies should specifically examine the impacts of newly implemented county policies that may affect the socioeconomic determinants of health behaviors, to determine whether the associations that we observed in this study may represent causal effects. For example, a recent systematic review of studies across international settings suggested that increased minimum wage policies reduce smoking [55]; additional work is needed to examine whether these results extend to recent county-level minimum wage increases or other similar policies in the U.S.

Our study suggests that the choice of methodology may be driving some of the inconsistencies in the existing literature in this field. The prior literature has relied primarily on statistical methods similar to our OLS models. These studies have been inconsistent, such that increased area-level disadvantage has been associated with both increased and decreased smoking and alcohol use, while others find no association [2, 6, 39, 56–58]. At the same time, prior studies using FE and marginal structural models have found persistent associations of area-level poverty with smoking and alcohol use [36, 37]. Randomized studies in this field are challenging due to logistical and ethical difficulties, although a handful exist. One randomized study found that poor individuals assigned to low-poverty neighborhoods had lower rates of short-term alcohol abuse [59], while another found no long-term impacts on risky healthy behaviors among youth whose families were randomly assigned housing vouchers [60]. Unsurprisingly, a recent systematic review found that the research on place-based effects on health behaviors is inconclusive [33]. Our findings suggest that future meta-analyses should pay special attention to the methods of included studies as a way of explaining contradictory findings.

Our study has several strengths. We use more rigorous longitudinal statistical techniques—i.e., fixed effects models—to overcome the confounding and reverse causation present in prior work in this field. Our use of a nationally representative data set also means that our results are more generalizable than prior studies that examined limited geographic areas. We also provide evidence on the effects of county-level measures of disadvantage, which are less frequently examined relative to place-based studies of smaller or larger geographic areas (e.g., U.S. census tracts or states). Relatedly, public health research departments and foundations have begun to support initiatives like the County Health Rankings to create metrics of county-level differences in health disparities [61], recognizing the importance of county-level determinants of population health.

Our study has several limitations. First, there may be measurement error in self-reported individual characteristics, as well as reporting biases related to frequency of substance use. Second, while both OLS and FE models adjust for time-varying confounding on observed characteristics, there may be confounding on unobserved factors; these might include time-varying aspects of individual or family socioeconomic status not captured by existing variables, or time-varying county and state characteristics that might influence both county disadvantage and individual health (e.g., minimum wage policies or alcohol prices). Consequently, we would not interpret these findings as causal estimates. Nevertheless, FE models represent an improvement over standard OLS modeling techniques, which fail to consider time-invariant confounding and which have dominated the area effects literature [62]. Also, county-level socioeconomic measures beyond the three we examined here are generally not available during this time period for the entire country; however, future studies could seek to compile a richer set of county-level predictors. Finally, one can imagine many interventions to improve health behaviors by addressing individual- and county-level disadvantage, representing a violation of the consistency assumption in causal inference. Absent an exogenous intervention or natural experiment, observational studies can only obliquely inform such strategies [63]. Nevertheless, this avenue of research should be considered one component of a pluralistic approach to triangulate the effects of place-based factors on health [64].

## Conclusions

Our findings highlight the challenge in disentangling the effects of county-level socioeconomic disadvantage on risky health behaviors, suggesting that methodological differences may explain some of the inconsistencies in the existing literature in this field. Few studies have implemented multiple statistical methods to disentangle these complex relationships. It is rare that place-based exposures can be randomized, and consequently, there is sparse inconsistent evidence that policymakers and advocates might use to design interventions to address the contextual determinants of risky health behaviors. While some have called for greater reliance on experimental studies [65], these are typically expensive and logistically or ethically unfeasible. Alternatives include increased attention to the use of more rigorous statistical methods and the identification of natural experiments, some of which suggest that area-level socioeconomic disadvantage influences health outcomes [66]. With the increasing availability of longitudinal and linked data, we are hopeful that our study contributes to a greater understanding of these pathways to guide future interventions.

## Additional files

Additional file 1: **Table S1**. Ordinary Least Squares Analysis of the Association between Lagged County-Level Characteristics and Individual Health Behaviors, U.S. National Longitudinal Study of Youth, 1992–2012. (DOCX 23 kb)

Additional file 2: **Table S2**. Fixed Effects Analysis of the Association between Lagged County-Level Characteristics and Individual Health Behaviors, U.S. National Longitudinal Study of Youth, 1992–2012. (DOCX 23 kb)

## Abbreviations

FE: Fixed effects
NLSY: National Longitudinal Survey of Youth
OLS: ordinary least squares
