# Supplementary material for: The association of county-level socioeconomic factors with individual tobacco and alcohol use: a longitudinal study of U.S. adults
Source: BMC Public Health. 2019 Apr 11;19:390. doi: 10.1186/s12889-019-6700-x (PMC6458796; doi:10.1186/s12889-019-6700-x)
Supplement: Supplementary file 1 — Table S1. Ordinary Least Squares Analysis of the Association between Lagged County-Level Characteristics and Individual Health Behaviors, U.S. National Longitudinal Study of Youth, 1992–2012. (DOCX 23 kb) [file 12889_2019_6700_MOESM1_ESM.docx]

**Additional file 1: Table S1. Ordinary Least Squares Analysis of the Association between Lagged County-Level Characteristics and Individual Health Behaviors, U.S. National Longitudinal Study of Youth, 1992-2012**

|  | **β Coefficient [95% CI]** | | | |
| --- | --- | --- | --- | --- |
|  | Smoker | Daily Cigarettes | Binge Drinking | Daily Drinks |
| County characteristics |  |  |  |  |
| Unemployment rate | -0.0052* | -0.083* | -0.00011 | 0.000058 |
|  | [-0.0077, -0.0027] | [-0.13, -0.033] | [-0.0024, 0.0021] | [-0.013, 0.013] |
| Income (per $1,000) | -0.00066* | -0.028* | -0.00064* | -0.0027* |
|  | [-0.0014, 0.000092] | [-0.041, -0.015] | [-0.0012, -0.000067] | [-0.0056, 0.00020] |
| % < high school | 0.00023 | 0.0089 | 0.00095* | 0.0045* |
|  | [-0.00058, 0.0010] | [-0.0039, 0.022] | [0.00023, 0.0017] | [0.00064, 0.0084] |
| Female | -0.022* | -0.70* | -0.15* | -0.83* |
|  | [-0.038, -0.0056] | [-1.00, -0.40] | [-0.17, -0.14] | [-0.89, -0.76] |
| Race (ref white/other) |  |  |  |  |
| Black | -0.047* | -2.74* | -0.064* | -0.51* |
|  | [-0.066, -0.027] | [-3.10, -2.39] | [-0.078, -0.051] | [-0.58, -0.43] |
| Hispanic | -0.12* | -3.62* | -0.013 | 0.056 |
|  | [-0.14, -0.096] | [-4.01, -3.23] | [-0.030, 0.0036] | [-0.048, 0.16] |
| Ln(Household income) | -0.010* | -0.10* | 0.0066* | 0.060* |
|  | [-0.014, -0.0068] | [-0.16, -0.046] | [0.0047, 0.0085] | [0.048, 0.072] |
| Education (ref < HS) |  |  |  |  |
| High school | -0.16* | -2.81* | -0.017* | -0.17* |
|  | [-0.18, -0.13] | [-3.37, -2.25] | [-0.035, 0.0011] | [-0.29, -0.045] |
| Some college | -0.22* | -4.03* | -0.057* | -0.37* |
|  | [-0.25, -0.19] | [-4.58, -3.47] | [-0.077, -0.038] | [-0.50, -0.25] |
| College or more | -0.36* | -6.57* | -0.096* | -0.56* |
|  | [-0.39, -0.34] | [-7.14, -6.01] | [-0.12, -0.076] | [-0.68, -0.43] |
| Married | -0.076* | -1.00* | -0.054* | -0.39* |
|  | [-0.095, -0.057] | [-1.34, -0.66] | [-0.068, -0.039] | [-0.47, -0.31] |
| Ln(Weeks unemployed) | 0.0033* | 0.051* | 0.00092* | 0.0066* |
|  | [0.0026, 0.0040] | [0.039, 0.064] | [0.00039, 0.0015] | [0.0032, 0.010] |
| Number of children | -0.016* | -0.24* | -0.010* | -0.036* |
|  | [-0.022, -0.0098] | [-0.35, -0.14] | [-0.015, -0.0058] | [-0.062, -0.0095] |
| No. Observations | 39,706 | 37,680 | 36,965 | 41,511 |
| No. Individuals | 9,052 | 8,992 | 8,718 | 8,864 |
| * p < 0.05. HS = high school. County-level characteristics included annual unemployment rate, inflation-adjusted per capita personal income, and percent with less than a high school education, measured during the survey wave prior to the measurement of the outcome. Analyses were conducted using multivariable linear regressions with imputed data. Linear probability models were used for binary outcomes. Additional controls included fixed effects for year. | | | | |
